# Learning signs with NAO: humanoid robot as a tool for helping to learn Colombian Sign Language

**DOI:** 10.3389/frobt.2024.1475069

**Published:** 2024-11-14

**Authors:** Juan E. Mora-Zarate, Claudia L. Garzón-Castro, Jorge A. Castellanos Rivillas

**Affiliations:** ^1^ Engineering Faculty, Research Group CAPSAB, Universidad de La Sabana, Chía, Colombia; ^2^ Engineering Faculty, Universidad de La Sabana, Chía, Colombia

**Keywords:** Colombian Sign Language (CSL), neural networks, machine learning, social robots, education, human-robot interaction (HRI)

## Abstract

Sign languages are one of the main rehabilitation methods for dealing with hearing loss. Like any other language, the geographical location will influence on how signs are made. Particularly in Colombia, the hard of hearing population is lacking from education in the Colombian Sign Language, mainly due of the reduce number of interpreters in the educational sector. To help mitigate this problem, Machine Learning binded to data gloves or Computer Vision technologies have emerged to be the accessory of sign translation systems and educational tools, however, in Colombia the presence of this solutions is scarce. On the other hand, humanoid robots such as the NAO have shown significant results when used to support a learning process. This paper proposes a performance evaluation for the design of an activity to support the learning process of all the 11 color-based signs from the Colombian Sign Language. Which consists of an evaluation method with two modes activated through user interaction, the first mode will allow to choose the color sign to be evaluated, and the second will decide randomly the color sign. To achieve this, MediaPipe tool was used to extract torso and hand coordinates, which were the input for a Neural Network. The performance of the Neural Network was evaluated running continuously in two scenarios, first, video capture from the webcam of the computer which showed an overall F1 score of 91.6% and a prediction time of 85.2 m, second, wireless video streaming with NAO H25 V6 camera which had an F1 score of 93.8% and a prediction time of 2.29 s. In addition, we took advantage of the joint redundancy that NAO H25 V6 has, since with its 25 degrees of freedom we were able to use gestures that created nonverbal human-robot interactions, which may be useful in future works where we want to implement this activity with a deaf community.

## 1 Introduction

**FIGURE 1:**
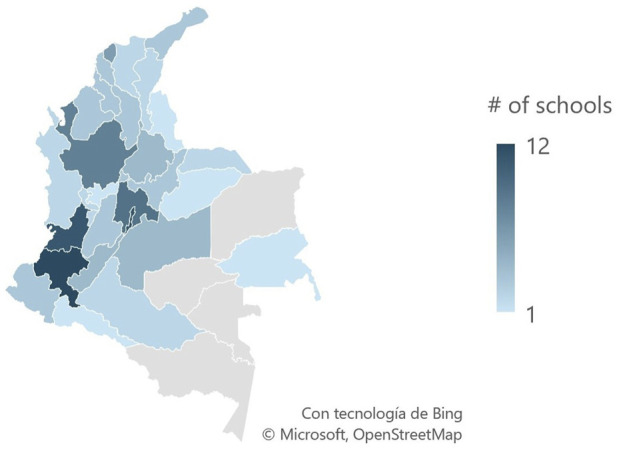
Number of schools offering bilingual bicultural education in Colombia by department.

People with disabilities constitute 16% of the global population, with 31% experiencing hearing problems, which significantly impacts their quality of life (World Health Organization, 2023; World Health Organization, 2024). In Colombia, where around 483000 people have hearing impairments, access to education is limited, with only one Colombian Sign Language (CSL) interpreter for every 1,152 deaf individuals and just 36% of interpreters working in basic education (Instituto Nacional Para Sordos, 2020). Moreover, out of more than 75,000 educational institutions in the country, only 109 offer bilingual and bicultural education, and some regions lack from CSL institutions as seen in Figure 1 (Instituto Nacional Para Sordos, 2024).

**FIGURE 2:**
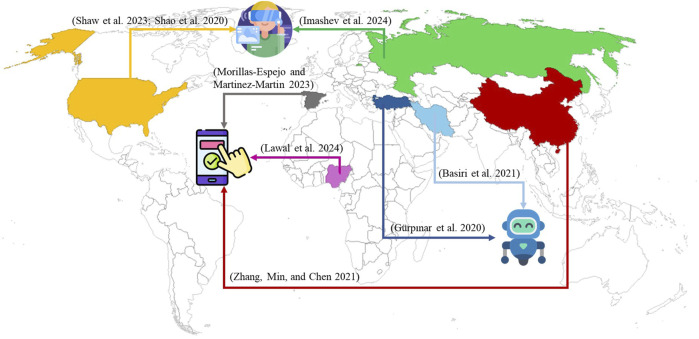
Prescence of different technologies for teaching sign languages.

To address this issue, data gloves and computer vision (CV) technologies have been the most frequent developed for sign language recognition, achieving performance levels above 90% as the recent studies in Table 1 show. However, both have their limitations: data gloves cannot predict the position of the hands relative to the body (Liang et al., 2024), while CV approaches require high computational resources (González-Rodríguez et al., 2024). Sign language recognition technologies have also been integrated into sign language translation systems (Teran-Quezada et al., 2024; Jintanachaiwat et al., 2024; Shahin and Ismail, 2024) and educational tools such as social robots (Gürpınar et al., 2020), mobile applications (Zhang et al., 2021), and Virtual Reality (Shao et al., 2020), all of which have proven effective in enhancing sign language learning and enabling these tools to be adopted by some other sing languages. However, as shown in Figure 2, the development and implementation of these technologies are concentrated in less than the 4% of the countries. Despite the promising results that may help to mitigate the gap in access to education for the deaf population, progress remains scarce in countries like Colombia. Works such as those of Pereira-Montiel et al. (2022); Gonzalez et al. (2024) are the most recent in the translation of CSL sentences and words, but there is still a gap on developing educational tool for this sign language.

**TABLE 1:** Recognition accuracies for sign language detection techniques.

Technique	Sign Language	Recognition accuracy	References
Computer Vision + Machine Learning	Japanese	96%–99%	Kondo et al. (2024)
Computer Vision + Machine Learning	Indonesian	94%–96%	Ilham et al. (2024)
Sensory Glove + Machine Learning	American	94%	Pal et al. (2024)
Sensory Glove + Machine Learning	Chinese	97%–98%	Ji et al. (2023)

In Colombia, early taught vocabulary in CSL includes topics like greetings, pronouns, numbers, and colors (Universidad de Bogota Jorge Tadeo Lozano, 2024; Universidad de Los Andes, 2024; Universidad ECCI, 2024). Therefore, this study proposes a performance evaluation for an educational tool capable of recognize all the 11 color-based signs in the CSL. To achieve this, we utilized the humanoid robot NAO H25 V6, which features 25 degrees of freedom and can engage in human-like interactions through the execution of gestures. Additionally, the education tool is enhanced with RGB eye LEDs and tactile buttons to provide both visual and physical feedback. This setup may help learners quickly assess their performance during the activity and maintain their engagement. Furthermore, NAO has shown effective as an educational tool that engages users through interactive experiences (Kurtz and Kohen-Vacs, 2024; Cruz-Ramírez et al., 2022; Mutawa et al., 2023). The proposed sign recognition system consists of the design and training of a Recurrent Neural Network (RNN), that uses as input hand and torso landmarks extracted with MedaPipe from a parallel video capture process. When validating the Neural Network (NN) performance, was found an F1-Score above the 90%, however, was also identified, that the latency needs to be improved when continuous sign recognition from the NAO H25 V6 camera. As future work, the range of sign topics recognized by the educational tool will be expanded to include a broader selection of early-taught vocabulary in CSL.

## 2 Materials and methods

### 2.1 Neural network design

**FIGURE 3:**
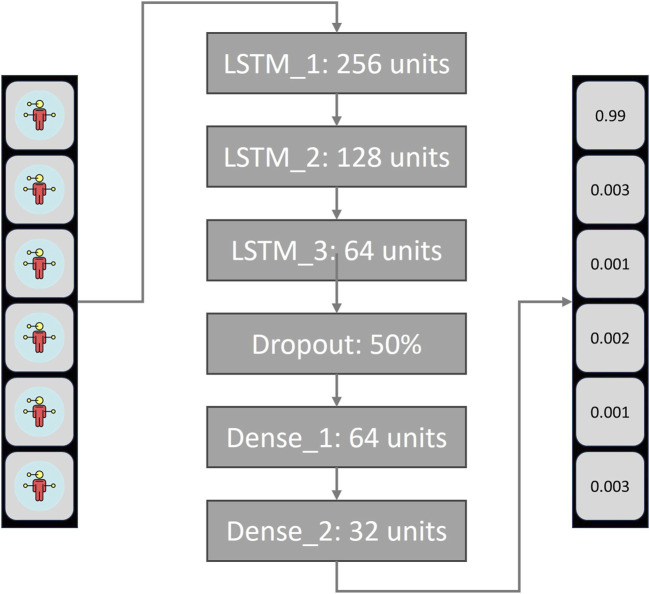
Neural Network architecture for dynamic sign detection.

A RNN of the Long-Term Short-Term Memory (LSTM) type was built, because when working with time-dependent information, such as dynamic or moving signs, an architecture that preserves information from the past is needed to make the classifications (See Figure 3). The NN architecture consists of a sequential model with three stacked LSTM layers of 256, 128, and 64 units, respectively, using ReLU activation, followed by a dropout layer (0.5). It then has three dense layers with 64, 32, and 11 units, where the final layer uses SoftMax activation for multi-class classification, finally, when training, a stochastic gradient descent method was used for the optimization (Adam) and a loss function for multi-class classification models (categorical cross entropy) where used. The NN obtained an F1-Score of 92% and was evaluated under a training-test split in the ratio 80–20, with a dataset for the CSL colors (white, brown, gray, purple, orange, black, red, pink and green). The data was obtained from videos of 20 signers with a length of 30 frames and were post-processed to extract with the MediaPipe tool the coordinates of the torso and right hand. It is important to clarify that the training and test set did not share people, to avoid biases when training and to guarantee that the NN was able to detect signs of new users.

### 2.2 Sign detection system

**FIGURE 4:**
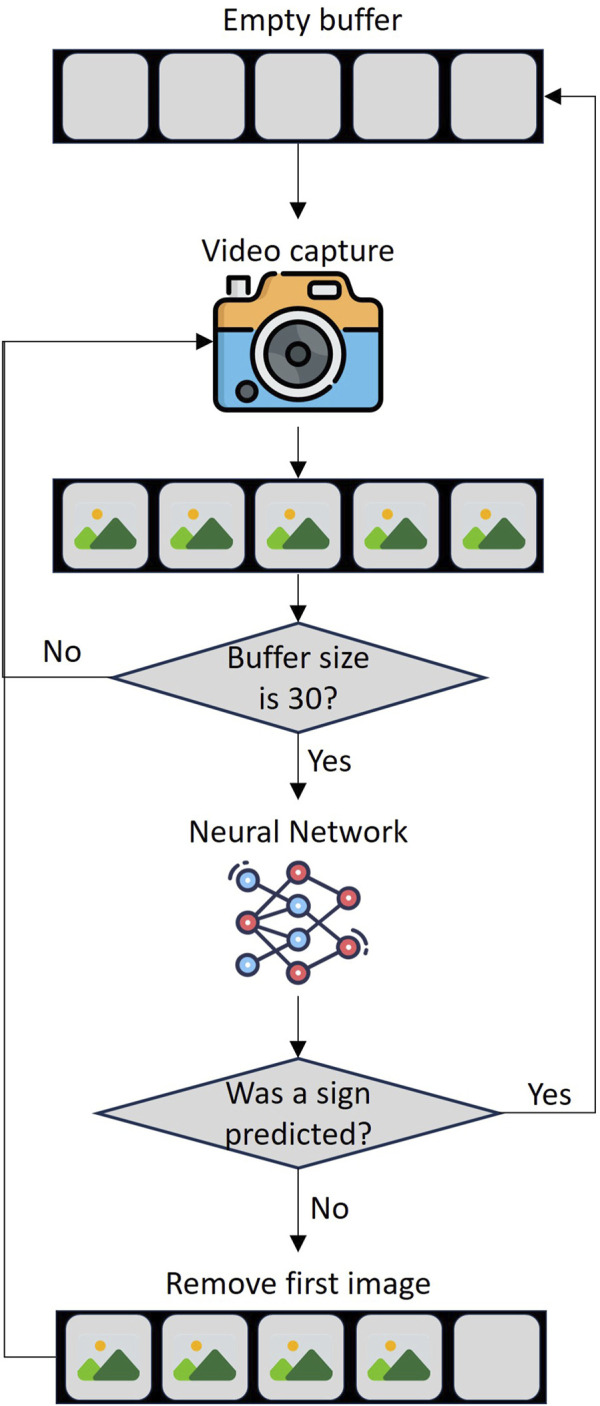
Continuous sign language detection process.

The sign detection system operates in a Python 3.12.3 script that runs on a laptop equipped a Core i7 8665U processor and 8 GB of RAM was made. The code has two simultaneous processes, the first one is capturing a video signal from the integrated webcam of the laptop which it will take images every 4 frames and then by means of the MediaPipe tool create a list with the 54 coordinates of the torso. The second process fills a data buffer with the list created in the first process, when the buffer reaches a size of 30 lists, it enters the NN to make the prediction, if it predicts with a tolerance higher than 99%, the buffer is emptied because a word has already been detected (see Figure 4). On the other hand, when capturing video from the NAO H25 V6, the processes will not run simultaneously, it will first capture video by a designed activity (See Section 2.4) and send it to the Python 3.12.3 script via TCP/IP communication.

### 2.3 Neural network evaluation

**FIGURE 5:**
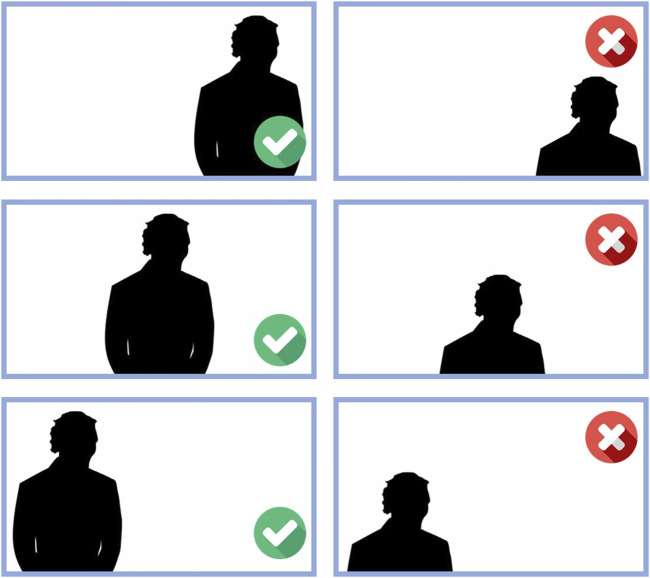
Description of how the user should be in frame for the sign detection process.

When evaluating a classification algorithm that is going to run continuously, it is crucial to assess both its speed and accuracy. For speed, timestamps were used when the buffer fills and empties. To evaluate accuracy, three tests performing each sign 10 time with a rest between signals of 1–2 s was made. The choice of 3 experiments with a sample size of 10 each was based on the binomial proportion formula, considering an expected 5% performance drop from results on the testing data and a theorical performance of 99% due to the higher results found in literature. Those considerations led to a calculated sample size of 29, that was divided into three experiments to prevent user fatigue. Additionally, the test was performed ensuring that the user was framed as shown in the first column of Figure 5, since the NN was trained under these same conditions. Also, there was a cold Light Emitting Diode (LED) lightning in a room where no direct light from outside enters, to ensure that the MediaPipe tool would always detect the coordinates of the torso and hand.

### 2.4 Actions performed by NAO H25 V6

Unlike robotic platforms such as mobile or industrial ones, social robots such as the NAO H25 V6 robot (SoftBank Group Corp., Japan) manage to have more friendly interactions with humans, because they can give the appearance that they are expressing an emotion (Hellou et al., 2023). For this reason, the NAO H25 V6 was controlled by means of a Python 2.7.18 code, which with the import of the SDK 2.8.6 accessed the modules shown below:

• ALLeds: Control of LED’s
• ALVideoRecorder: Video recording
• ALTouch: Reading capacitive sensors
• ALRobotPosture: Set predefined positions
• ALAnimationPlayer: Execute predefined movements
• ALMotion: Joint control

**FIGURE 6:**
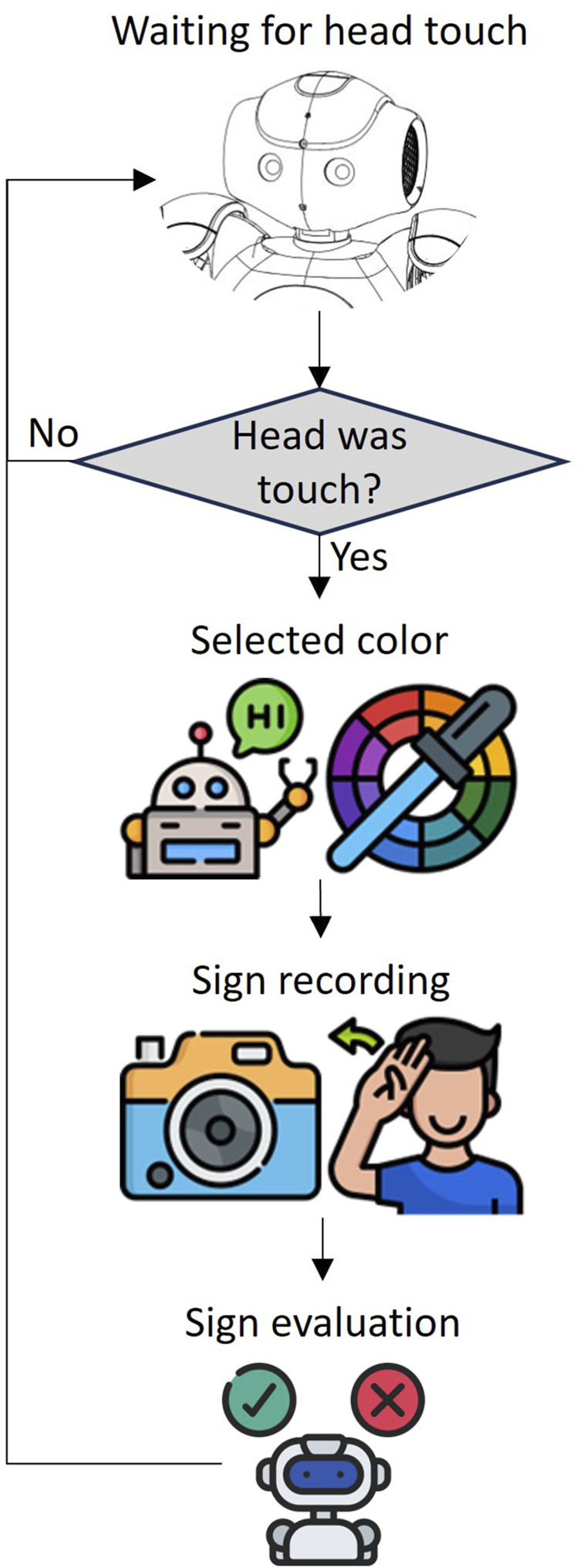
Activity with NAO H25 V6 for evaluating a sign.

The modules allowed the development of an activity as described below (see Figure 6).

1. Starting position: The NAO H25 V6 is placed in a standing position and turns its head forward up to 4.5°. This is because the robot is located on a table of 1.1 m high and the user is at approximately 1.5 m.
2. Start of the activity: The user can choose between 2 evaluation modes. The first one will be activated by pressing the first touch button on the head of the NAO H25 V6. This will allow to select any of the 11 colors by means of a touch menu that will change selection each time the first button on the head of the robot is pressed. The second mode will be activated by pressing the second button on the head of the NAO H25 V6, and the color will be randomly selected. Once the color is selected, the robot greets with the gesture “animations/Stand/Gestures/Hey_1”and lights up its eyes in the color of the sign that should be made.
3. Recording of the signal: Recording is at a rate of 8 Frames Per Second (FPS) for 4 s and starts after the LEDs of the robot’s eyes blink 3 times, once in the recording state the eight LEDs of each eye are turn on and will be turn off progressively as the 4 s of recording are over, thus giving feedback to the user of the remaining time.
4. Prediction of the signal: The video is stored in the NAO H25 V6 memory and is sent via TCP/IP to a second code in Python 3.12.3 that is responsible for making the prediction and respond with the color of the executed signal.
5. Result: With the received message, the robot lights up its eyes with the color that the user made. In addition, if the signer made the sign properly, the NAO H25 V6 affirms by making the gesture “animations/Stand/Gestures/Yes_2”, or otherwise, denies by making the gesture “animations/Stand/Gestures/No_9”.

## 3 Results

### 3.1 Neural network evaluation

**FIGURE 7:**
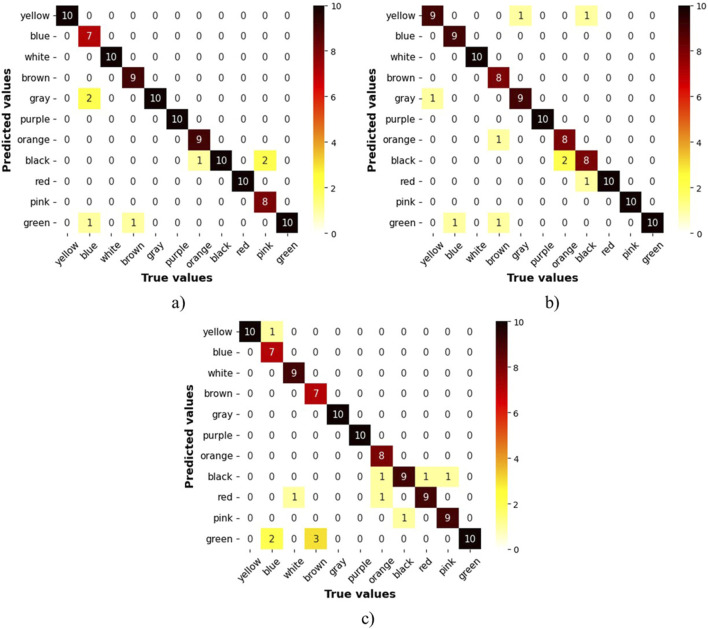
Confusion matrix for sign detection when using a webcam for video capture. **(A)** 1st trial, **(B)** 2nd trial, **(C)** 3rd trial.

**FIGURE 8:**
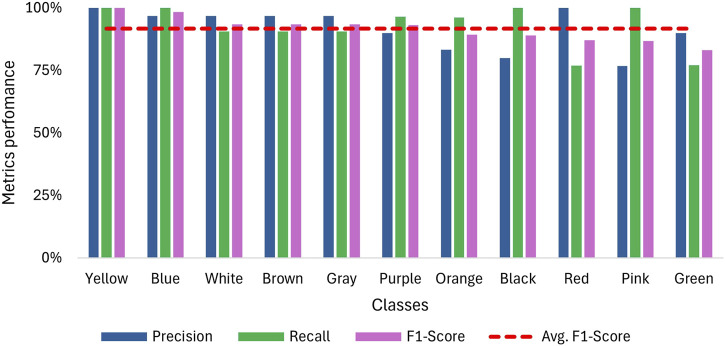
Overall recall, precision and F1-Score for sign detection when using a webcam for video capture.

As mentioned in the methodology, each class is considered as a binomial problem in which three tests with a sample size of 10 were conducted on the NN to evaluate its performance. The results for accuracy are shown in Figure 7, where the confusion matrix illustrates that most predictions fall within the true positive and true negative zones. The lowest data point in this zone is 7, corresponding to a categorical accuracy of 70% for that class. Also, Figure 8 displays the average metrics across the three tests, with the purple sign achieving a F1-Score of 100%, which means it was always recognized, and no sign was confused with it. The F1 scores for other signs range from 98.3% to 83.1%, resulting in overall metrics of 91.6% for the F1 score, 92.6% for recall, and 91.5% for accuracy. Additionally, the precision is more affected than recall when the F1-score decreases, suggesting that the NN has more difficulty with precision than recall.

**FIGURE 9:**
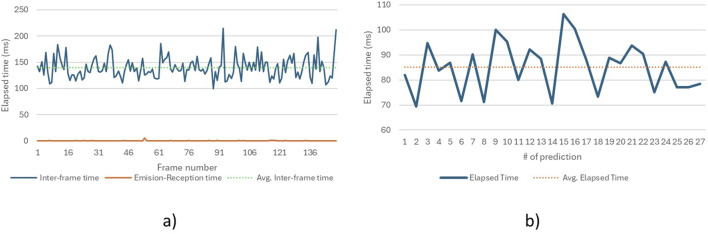
Timestamps for the sign recognition system when using a webcam for video capture **(A)**. Elapsed time between frames and script processes **(B)**. Elapsed time for the model to make a prediction.

On the other hand, Figure 9A shows the time required by the algorithm to process each image using MediaPipe and send it to the sign prediction process, with an average time of 139.8 m per image for 150 frames. This translates to a frame rate of 28.6 FPS, which represents only a 4.7% reduction compared to the camera’s capture rate of 30 FPS. Additionally, Figure 9A displays the time it takes for the algorithm to send data from the first process and capture it in the second, averaging 0.2 m between data emission and reception, ensuring no synchronization issues between processes. Finally, Figure 9B shows the average prediction time of 85.2 m for 27 predictions, indicating that processing the 30-frame buffer is faster than the rate of incoming data.

### 3.2 Activity with the robot NAO H25 V6

**FIGURE 10:**
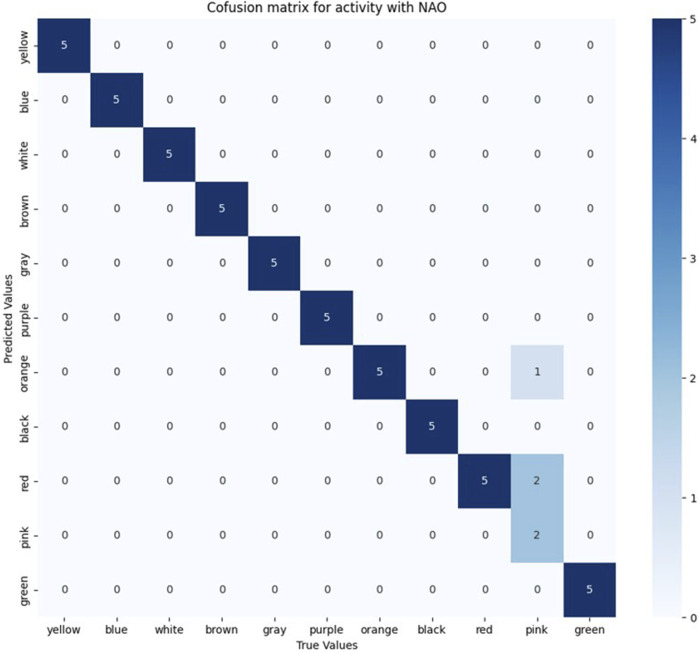
Confusion matrix for sign detection when using NAO H25 V6 camera for video capture.

**FIGURE 11:**
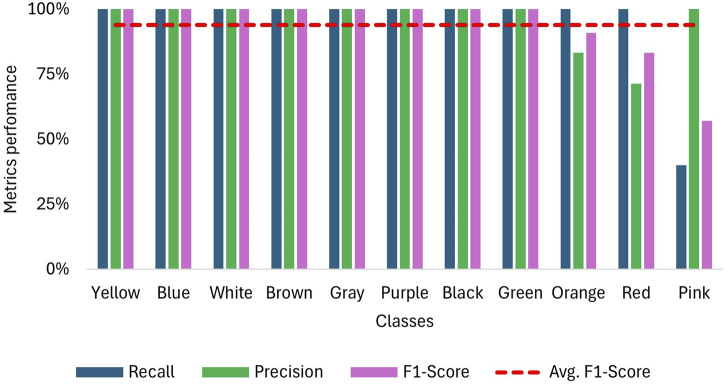
Recall, precision and F1-Score for sign detection when using NAO H25 V6 camera for video capture.

Five trials were conducted for each color sign, with the only prediction error occurring for the pink sign, which, as shown in the confusion matrix in Figure 10, was confused twice with red and once with orange. Furthermore, as Figure 11 shows, 8 signs achieved an F-1 score of 100% and for the colors red, orange and pink, F-1 scores of 90.9%, 83.3% and 57.1% were obtained respectively. These results translate into an overall F-1 score of 93.8%.

**FIGURE 12:**
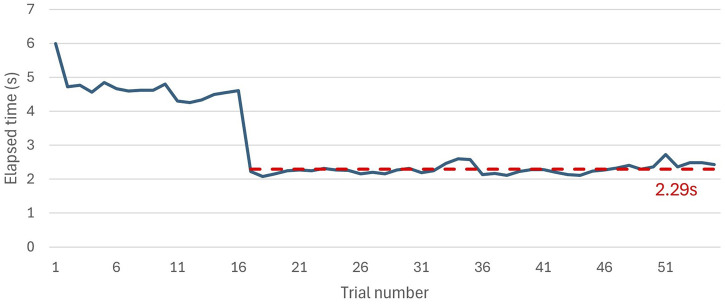
Elapsed time between the end of NAO H25 V6 recording the video and lightning up his eyes with the predicted color.

On the other hand, the response time between the end of recording and when the NAO H25 V6 illuminates its eyes with the predicted sign color was also measured. As shown in Figure 12, the initial iterations took longer; however, after the model passed its warm-up stage, the response time decreased to an average of 2.29 s.

## 4 Discussion

This paper shows the use of the NAO robotic platform developing that could supports de CSL learning. For it, a NN that executes on a computer was design for running continuously in two different scenarios, video capture from the integrated computer webcam and wireless streaming from the NAO H25 V6 camera. The NN detection capability and latency were evaluated, finding promising results in sign detection accuracy for both scenarios, and low latency for the webcam scenario. However, latency remains a limitation when using the NAO H25 V6, as it is 2.2 s slower than the webcam scenario. This issue arises due to the MediaPipe preprocessing, which is not done in parallel with the video capture, since the sign detection script only processes images after recording is completed. To address this, future works, should aim to run all operations in the NAO H25 V6 computer and use parallel processing to improve latency. Additionally, it would be relevant to increase the number of signs that the NN is able to detect, to design activities that involve the construction of sentences and not only the handling of words.

On the other hand, Table 2 provides a comparison of related studies that involve the use of humanoid robots for sign language recognition. For instance, Li et al. (2023) achieved 100% accuracy with a NN containing fewer than 3,000 parameters; however, it was only trained with two signers and could recognize only four signs, which limits its generalization to new users. Similarly, Hosseini et al. (2024) utilized a custom humanoid robot capable of mimicking various signs, thanks to its five-fingered hands with independent motors. Their focus, however, was on recognizing and imitating signs using a data glove, while our work demonstrates the novelty of using humanoid robots as a tool to support sign language education. Additionally, we leveraged the robot’s camera for sign detection, with only the data processing occurring externally.

**TABLE 2:** Use of humanoid robots for sign language imitation and/or recognition.

References	Li et al. (2023)	Hosseini et al. (2024)	Ours
Robot	NAO	RASA	NAO H25 V6
# of signs	4	16	11
# of signers in dataset	2	12	20
Sensor	Kinect + Myo-armband	DataGlove	RGB camera
# of Neural Network parameters	2,764	2,462,912	682,187
Feature map	1,450	600	4,860
Accuracy	100%	70%	93.8%

The studies summarized in Table 2 also highlight the importance of humanoid robots’ joint redundancy in mimicking and teaching signs. Flexibility in a robot’s joints is crucial for accurately mimicking signs, as finger position, hand orientation, and hand position relative to the torso are critical in sign language (Meghdari et al., 2019). Hosseini et al. (2024) addressed this with their RASA robot, which has 29 degrees of freedom in its upper torso. Redundancy is also significant in educational activities, as physical interaction with the robot enhances user engagement (Timmerman and Ligthart, 2023). In this study, we aimed to foster user engagement through gestures and non-verbal interactions, alongside the use of buttons for tactile feedback.

The NN implemented in this study was based on an LSTM architecture, demonstrating its potential in sign detection systems without being constrained by processing limitations. This opens opportunities to work with deeper NNs in the future, capable of classifying a greater variety of signs. MediaPipe landmarks proved to be an effective tool for feature extraction that could outperformed CNN-based feature maps, which generate larger data per frame. For instance, the NN proposed Sincan and Yalim Keles (2020) outputs 131,071 features per frame, compared to just 162 features produced by MediaPpie. However, future work should consider enhancing this feature map by including joint angles and excluding irrelevant landmarks. As the confusion matrices on Figure 7 shows certain signs, such as blue and green, were confused due to similar movements but differing hand orientations. Including joint angles could provide more precise characteristics, reducing these types of errors.

Also, Table 3 compares studies that have explored continuous dynamic sign recognition across various sign languages. This study achieved an accuracy of 91.6%, which is comparable to other works that report accuracies above 90%. However, it stands out for its significantly lower computational cost, requiring only 35 m per frame, which is 93% less time than the study with the highest detection accuracy. This demonstrates the efficiency of the proposed system in terms of processing speed while maintaining competitive accuracy.

**TABLE 3:** Performance for continuous sign recognition models.

References	Rahaman et al. (2024)	Sharma et al. (2024)	Joshi and Patel, (2024)	Ours
Accuracy	95.43%	99.7%	95.45%	91.6%
Computational cost (ms/frame)	56	500	-	35
Sign Language	Bangala	Indian	Indian	Colombian
# of signs	100	12	59	11

Additionally, it is important to highlight some of the technical limitations found in terms of detection capability and latency. Regarding detection, the system was prone to making incorrect predictions when the user was not performing any signs. As for the algorithm’s latency, it increased by 2.2 s when making a wireless connection to the NAO H25 V6 camera from the computer. Although this latency is acceptable in terms of computational performance, further studies are needed to determine whether this delay might affect user interaction.

Since the objective of this work is to support CSL teaching for both hearing and non-hearing individuals who want to learn this language, the first step was to conduct a performance evaluation of the proposed educational tool. This evaluation demonstrated reliability in terms of sign detection accuracy and latency in a controlled environment, as presented in this paper’s results. Based on these findings, the next step is to test the tool with real users to explore if features such as non-verbal interactions and visual feedback provided by the NAO H25 V6, are perceived as effective and engaging by the users, also, we aim to determine if the latency impacts the learning experience. To achieve this, the tool will be tested at both a bilingual bicultural school (secondary education) and at the Universidad de La Sabana (higher education), with the intention of reporting the results in a coming paper. Finally, in the future, we aim to expand the set of signs that the system can recognize by integrating additional vocabulary such as greetings, pronouns, and numbers, as experts suggest these are the primary topics taught in the early stages of CSL learning (Universidad de Bogota Jorge Tadeo Lozano, 2024; Universidad de Los Andes, 2024; Universidad ECCI, 2024).

## 5 Conclusion

An activity was designed to support the teaching of CSL using the NAO H25 V6 robotic platform. For sign detection we used the robot’s upper camera that records the user executing a sign, we also took advantage of the redundancy in joints that the robot has, to use human-like gestures which allow non-verbal human-machine interactions and can be useful when working with deaf people. In addition, the integration of tactile buttons allows to have a physical interaction with the robot, which with gestures and LEDs integration can allow the user to feel more involvement of the robot in the activity. Likewise, a RNN of the type LSTM was built for the detection of 11 colors of the CSL, the architecture has three LSTM and dense layers with 682187 parameters. Where promising results were obtained for sign detection accuracy and computational cost, with an F1-Score of 91.6% and response time of 35 m (28.6 FPS) per frame for the test using a webcam, while the test using the NAO camera through a TCP/IP connection obtained results with an F1-Score of 93.8% and 2.29 s to predict a sign. Finally, as future work, we will run all the processes on the NAO computer to improve the response time per sign and we hope to take this activity to a bilingual bicultural school; to measure the impact, it would have on people who are learning basic CSL vocabulary.

## Data Availability

The raw data supporting the conclusions of this article will be made available by the authors, without undue reservation.
